# The challenge of optimising ablation lesions in catheter ablation of ventricular tachycardia

**DOI:** 10.1002/joa3.12489

**Published:** 2020-12-28

**Authors:** Riccardo Proietti, Luca Lichelli, Nicolas Lellouche, Tarvinder Dhanjal

**Affiliations:** ^1^ Department of Cardiology University Hospital Coventry & Warwickshire NHS Trust Coventry UK; ^2^ University of Warwick (Medical School) Coventry UK; ^3^ Department of Cardiac, Thoracic, Vascular Sciences University of Padua Padua Italy; ^4^ Hopital Henri Mondor Albert Chenevier Creteil France; ^5^ Inserm U955 University Paris Est Creteil Paris XII Paris France

**Keywords:** catheter ablation, long‐term outcome, multipolar mapping, omnipolar mapping, ventricular tachycardia

## Abstract

Radiofrequency catheter ablation has become an established treatment for ventricular tachycardia. The exponential increase in procedures has provided further insights into mechanisms causing arrhythmias and identification of ablation targets with the development of new mapping strategies. Since the definition of criteria to identify myocardial dense scar, borderzone and normal myocardium, and the description of isolated late potentials, local abnormal ventricular activity and decrementing evoked potential mapping, substrate‐guided ablation has progressively become the method of choice to guide procedures. Accordingly, a wide range of ablation strategies have been developed from scar homogenization to scar dechanneling or core isolation using increasingly complex and precise tools such as multipolar or omnipolar mapping catheters. Despite these advances long‐term success rates for VT ablation have remained static and lower in nonischemic than ischemic heart disease because of the more patchy distribution of myocardial scar. Ablation aims to deliver an irreversible loss of cellular excitability by myocardial heating to a temperatures exceeding 50°C. Many indicators of ablation efficacy have been developed such as contact force, impedance drop, force‐time integral and ablation index, mostly validated in atrial fibrillation ablation. In ventricular procedures there is limited data and ablation lesion parameters have been scarcely investigated. Since VT arrhythmia recurrence can be related to inadequate RF lesion formation, it seems reasonable to establish robust markers of ablation efficacy.

## Graphical abstract

New mapping strategies for radiofrequency ablation of ventricular tachycardia have been developed so substrate‐guided ablation has become the method of choice to guide procedures. Also, many ablation strategies have been developed using increasingly complex and precise tools. However long‐term success rates for VT ablation have remained static. Many indicators of ablation efficacy have been developed but in ventricular procedures ablation lesion parameters have been scarcely investigated. Since VT arrhythmia recurrence can be related to inadequate RF lesion formation, it seems reasonable to establish robust markers of ablation efficacy.

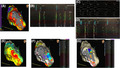

## 1 INTRODUCTION

Over the last decade, radiofrequency catheter ablation (RFCA) has become an established treatment for ventricular arrhythmias (VA). The recent international consensus report supports RFCA as a first‐line therapy for VA in normal heart Ventricular Tachycardia (VT) and in structural heart VT after ineffective anti‐arrhythmic drug (AAD) therapy.^1^ Several groups have taken this further, proposing prophylactic VT ablation before implantable cardiac defibrillator (ICD) insertion; however this remains an area of debate.^2^ Though substrate ablation prior to ICD implantation failed to show a significant reduction in VT recurrence, the studies demonstrated a reduction in both overall and cardiovascular mortality.^1^ The mechanism of this reduction in mortality despite persistent VT post‐ablation has yet to be elucidated.

The exponential increase in the treatment of VAs with RFCA in clinical practice has provided further insight into the mechanisms of these arrhythmias, improved identification of ablation targets with the development of new mapping strategies supported by mapping and ablation technological advances. Much of this growth in the field of VT RFCA has been focused on identification of the VT critical isthmus and localizing the site of origin of the arrhythmias.^3^

**FIGURE 1:**
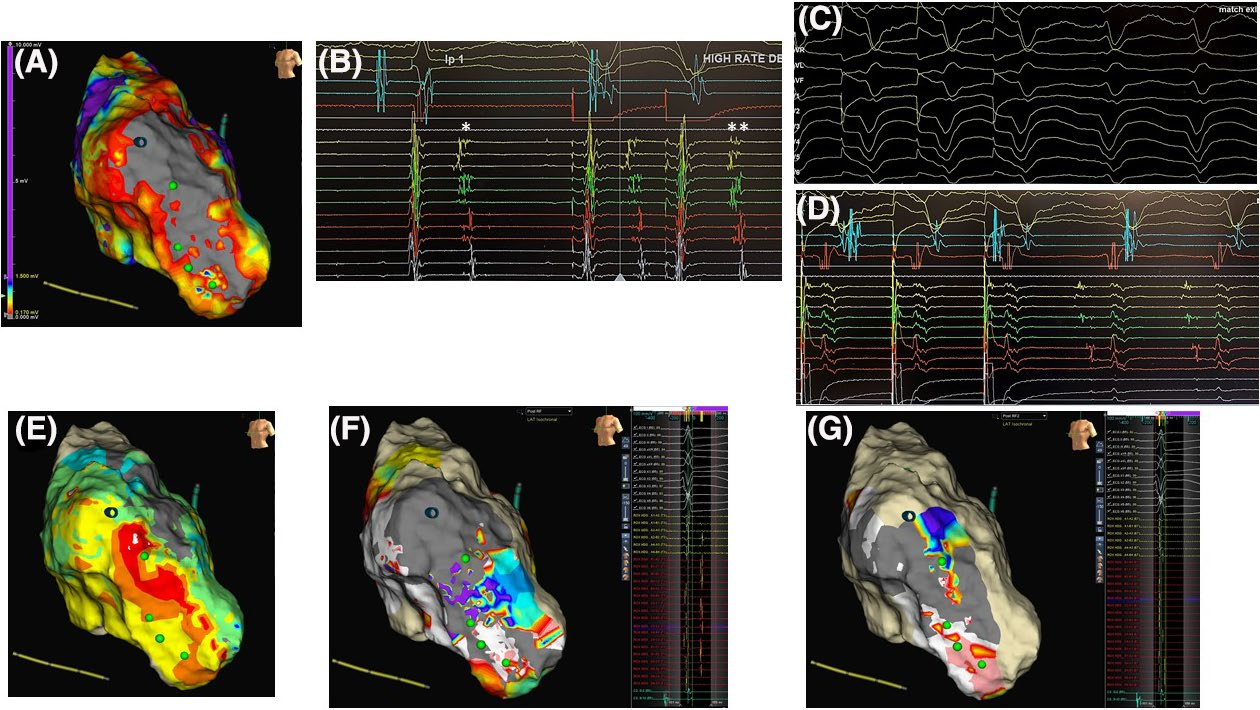
Patient with ischemic cardiomyopathy. VT tachycardia cycle length (TCL) 380 ms mapped using HD Grid. (A) Endocardial LV substrate map demonstrates anterior wall scar with heterogeneous scar extending towards the septum. Voltage scanning (0.17 mV) identifies a CC extending from the apical septal BZ into the dense scar region. Decrementing evoked potential (DEEP) potentials tagged green. (B) Example Late Potentials (single asterisk) DEEP potentials (double asterisks) identified with pacing from the right ventricular apex with two ventricular sensed extra stimuli at 400 ms (C) Pacemapping during entrainment mapping yields 12/12 pacemap from the apical septal BZ. (D) Entrainment from middiastolic potentials yields PPI‐TCL < 30ms with stim‐QRS > 60ms. (E) Clinical VT activation map: earliest activation at anterior dense scar region. (F) Postablation substrate remap shows residual late potentials still present (purple zone). (G) With further ablation residual late potentials completely eliminated

Traditionally, the 12 lead ECG has been a useful tool in defining the location of arrhythmia origin and this should not be neglected despite the subsequent development of highly specific complex mapping technologies. These technologies have helped to further define the VT site of origin from conventional entrainment and pacing maneuvers during triggered VT, towards substrate mapping during sinus or paced rhythm^4^, ^5^, ^6^; Substrate‐guided ablation has progressively become the method of choice to guide ablation^7^ achieving improved rates of intra‐procedural success and long‐term freedom from VT recurrences.^8^ The cornerstone of an effective substrate‐guided ablation strategy is the precise identification of diastolic conduction channels and abnormal fractionated electrograms (EGMs) that help to define potentially pro‐arrhythmic substrate within the borderzone (BZ) and dense scar region.^9^, ^10^, ^11^ We have combined these complementary mapping strategies into a strict mapping and ablation workflow (UHCW VT ablation workflow) as summarised in Figure 1.

After the first description by Marchlinsky et al, the electrical criteria for the characterization during substrate mapping of myocardial dense scar, BZ and normal myocardium have been universally adopted and more recently adapted by the EP community.^11^ The natural extension of this seminal work has been the quantitative and qualitative analysis of substrate electrograms (EGMs) which has provided further insights into the identification of slow diastolic conduction channels and abnormal electrical activity. This work extended from the appreciation of isolated late potentials (LP) to included local abnormal ventricular activity (LAVA)^12^ and more recently decrementing evoked potential (DEEP) mapping.^13^ Indeed, it has been shown that specific EGM signatures defining LPs with lower amplitude and shorter duration are greater predictors of the VT isthmus.^14^ Accordingly, a wide range of substrate ablation strategies have been developed over the years from scar homogenization to scar dechanneling or core isolation. Many of these topics have been the focus of a number of prior reviews, some published by our group.^15^, ^16^

Technological advances for VT mapping has been another field that has undergone exponential development. EGM amplitude for normal and abnormal tissue were described using the standard ablation and mapping catheters with 4‐mm tip electrodes, 1‐mm ring electrodes and 2‐mm interelectrode spacing. However, EGMs detected are strictly dependent on electrode size and orientation respective to wavefront propagation, which rapidly changes directions resulting in a changeable recording according to the vector line of assessment. As a result, these catheters may not identify low‐voltage EGMs propagating orthogonal to their orientation potentially missing critical arrhythmic substrate. More recently, multipolar electrode catheters with 1mm electrodes and variable interspacing have been introduced facilitating high‐density map construction. This enables an increase in the near‐field voltage detected and reduces far‐field signals providing a better definition of substrate. Furthermore, omnipolar mapping catheters also provide a wavefront directional assessment.^15^ This is advantageous in a structure like myocardial scar in which wavefront depolarization rapidly changes direction. Notably, the recently developed Advisor HD Grid Mapping Catheter (HD GRID) is a multipolar catheter with electrodes equally interspaced along each spline and arranged in a grid configuration^17^ The catheter will simultaneously record EGMs along and across different orthogonal vectors when point acquisition is set in the bidirectional WAVE configuration. This provides a more efficient point acquisition and greater VT substrate definition of the scar BZ so facilitating conduction channel identification and quantification, which are both important surrogates for ablation strategy.^16^ Our group has subsequently shown that VT mapping with the HD GRID has a significant positive impact on the long‐term outcomes of VT ablation (paper in press).

Indeed, an accurate, detailed substrate map plays a crucial role in short‐ and long‐term ablation efficacy as critical isthmus and slow conduction region identification is facilitated. These regions provide the substrate for reentrant circuits and are often characterized by the presence of isolated LPs^11^, ^18^, ^19^, ^20^, ^21^ that are used as indicators of the presence of a critical zone that is a target for ablation; in fact it has been demonstrated that LPs significantly increase the specificity for identifying the clinical VT circuit.^9^ Three zones within the myocardium are important to be recognized: “normal myocardium” defined as a voltage >1.5 mV, “dense scar” or low‐voltage myocardium defined by a voltage <0.5 mV and scar BZ or intermediate voltage myocardium with voltage between 0.5 mV and 1.5 mV.^11^, ^22^ It is important to recognise that with the advent of high‐density mulipolar mapping catheters, the bipolar voltage parameters that define BZ can range as low as 0.02 mV. Much of the focus is directed to scar BZ since initial studies in open‐heart models demonstrated that ischemic VT originates in the area surrounding the dense scar and operative removal of this tissue could cure 70%–80% of arrhythmias.^23^ More recent studies have partially confirmed this observation but have also highlighted that a significant proportion of reentrant circuit isthmuses exist within the dense scar, epicardium or in normal myocardium.^24^

Among the varying substrates for structural heart diseases an important determinant in ablation efficacy is the underlying cardiomyopathy: for instance the scar region in most ischemic cardiomyopathy (ICM) patients is endocardial^25^; in contrast, nonischemic dilated cardiomyopathy (NIDCM) usually presents as a patchy distribution of fibrosis largerly found in perivalvular region with less dense scarring^26^ and with more epicardial involvement. A study by Nakahara et al demonstrated that in NIDCM there is a significantly lower numbers of late potentials (<100 ms after the QRS) or very late potentials (>100 ms after the QRS) and a more epicardial distribution of fibrosis.^27^ They also demonstrated that in NIDCM there are fewer good‐to‐excellent pace‐match sites compared with ICM; moreover in NIDCM it has been demonstrated that there is a higher incidence of focal arrhythmia mechanisms.^28^ There is, however, evidence that a purely substrate‐guided ablation is an effective approach in the treatment of VT with lower success rates in NIDCM than ICM.^7^ Differences especially in long‐term outcomes may be because of the patchy nature of fibrotic burden in NIDCM and its epicardial distribution that make ablation more challenging; in fact it has been demonstrated by Dinov et al in the HELP VT study that combining endocardial and epicardial ablation especially in NIDCM substantially reduces long‐term recurrences of VT.^29^ Moreover there are inconclusive data regarding the predictors of ablation efficacy and long‐term survival free of VAs both in ICM and NICM.^30^, ^31^, ^32^, ^33^, ^34^, ^35^

The experience in ablation treatment of ventricular arrhythmias in normal hearts and more specifically in treatment of premature ventricular complexes (PVCs) has also advanced. It has been demonstrated that an ablative approach is more efficacious than conservative medical therapy with anti‐arrhythmic drugs (AADs).^36^, ^37^ Studies also demonstrated that PVC suppression may lead to reverse ventricular remodeling and amelioration of pump function in selected cases of PVC‐associated cardiomyopathy.^38^, ^39^, ^40^, ^41^, ^42^ On the other hand, there is debate as to whether this form of cardiomyopathy is cause or consequence of frequent PVCs.^41^, ^43^ For all these reasons a more precise risk‐benefit assessment was needed in order to decide if ablation is cost effective and it has been given by Jin‐sheng Wang et al^44^ who published a retrospective study in which they assessed predictors of complication and success in patients treated with PVC ablation. In a cohort of 1231 patients the overall success rate was 94.1% with a complication rate of 2.7% with left ventricular and epicardial (EPI) origins as predictors of complication, arguing that given an overall favorable prognosis if untreated,^45^ ablation is unadvisable in these origins.^44^

## 2 ABLATION MARKERS IN VT ABLATION

Embracing RFCA as an effective therapy for the treatment of VAs is based on the fundamental principle that the intervention delivers solid and durable ablation lesions in the human myocardium. RFCA utilises electromagnetic energy that once delivered at the myocardial tissue interface is transformed into heating that irreversible damages the viable myocytes causing the loss of cellular excitability. Heating to a critical temperature directly destroys the myocardium in contact with the catheter bipolar electrode delivering energy; this process is known as resistive heating.^46^ However considering the small dimension of the tip of the catheter, the size and width of the lesion is determined by the destruction of surrounding tissue by heating transfer, a process also known as conductive heating. Irreversible loss of cellular excitability generally occurs at temperatures exceeding 50°C, while at lower temperatures the damage is not permanent and myocytes can recover excitability. Indeed the clinical manifestation of the recovery of cellular viability is the recurrence of arrhythmias.^46^

Prior to the increasing application of RFCA in the ventricles, ablation therapy has been widely applied and studied in the atria and more specifically in the complex setting of atrial fibrillation (AF). There is a wealth of literature that has explored optimizing ablation lesions in AF to define the fine balance between the creation of durable ablation lesions versus the avoidance of lesion formation complications such as pulmonary vein stenosis, cardiac tamponade and collateral damage to adjacent structures such as the phrenic nerve and oesophagus. Due to the challenging nature of visualizing lesion formation in real time and ensuring an effective transmural lesion, different surrogate measures of lesion quality have been utilized. The fall in local impedance during ablation is considered as a marker of the direct effect of ablation in cardiac tissue.^47^, ^48^, ^49^

One of the major determinants of lesion formation is an adequate contact between the tip of the catheter and the myocardial surface. A major technological advancement in ablation catheters was the development of sensors at the distal tip capable of monitoring contact, contact force (CF). While the bulk of evidence has investigated the correlation between CF and outcome of pulmonary vein isolation (PVI) in AF, very limited data are available regarding VT ablation. Elsokkari et al reported the only experience so far showing that in a small cohort of patients with ischemic heart disease, a mean contact force of 10 g within the scar zone has the best correlation with effective lesion formation.^50^

A more recent ablation marker is the Force‐Time Integral (FTI), which multiplies CF by radiofrequency application duration; it has been demonstrated that it is predictive of PVI segment reconnection at repeat electrophysiology study.^51^ Limitations in this ablation parameter are the exclusion of maximal power settings being delivered and the assumption that a single target FTI is required in all myocardial segments with varying wall thickness and underlying substrate. Indeed, this partly explains that the usage of this marker is related to at least one PV reconnection in one‐third of AF ablation cases^52^; moreover the contribution of radiofrequency application duration is proportionally less important in lesion creation than CF especially for prolonged energy deliveries; in fact lesion depth and width are dependent on both power and ablation duration until it lasts more than 20s, beyond this duration, lesion characteristics depend only on power used^53^; so with the same FTI there is a potential large difference in lesion formation.

In order to overcome some of these important limitations the Ablation Index (AI) (Biosense Webster Inc) variable evolved which is calculated by incorporating power delivery in its formula and combining it with CF and time in a weighted equation. It has been shown to be a more precise estimation of lesion depth in a canine model.^54^ It has also been compared with FTI in a study by Das et al who demontrated that AI and FTI in a wide area circumferential ablation (WACA) segment are independently predictive of conduction recovery at follow‐up electrophysiology studies with a trend towards a greater sensitivity with AI. It was also highlighted that different myocardial regions have different minimum values of both indices required to prevent subsequent reconnections.^55^

Since its validation as a reliable marker of short‐ and long‐term ablation efficacy, increasing emphasis has been placed on AI, the feasibility in settings other than AF, and the pursuit to determine optimal cutoffs. In a recently published paper, Casella et al retrospectively investigated this topic in a multicenter observational study evaluating AI reliability in patients who underwent RFCA of idiopathic outflow tract PVCs performed using the CARTO electroanatomic mapping system. They investigated 1226 lesions and found that both maximum and mean AI values were significantly higher in patients without PVC recurrence even when stratified for anatomical areas. Lesions were analyzed according to acute and long time success and accordingly the AI values were lower both in patients with acute (usually ascribed to procedural reasons) and long‐term (6 months) failure; in this second case occasionally acute success was reached suppressing PVCs, but as a result of induced edema, recurrences manifested once the edema had resolved. Authors concluded that AI seems to be a reliable index to evaluate RFCA in PVC ablation^56^ and further explored the possibility to establish AI cutoffs. Despite the retrospective nature of the analysis which was not AI‐guided the authors proposed that maximum AI values were more reliable than mean AI values.

Unlike the field of AF ablation, where the AI algorithm has been studied extensively in the atrium,^57^ there is very limited experience on the utility of AI in catheter ablation of VT in patients with structural heart disease. In addition, although the ablation targets in scar‐related VT (areas of low‐voltage myocardium (LVM) and intermediate voltage myocardium (IVM)) have been described, data on ablation efficacy such as AI, CF, impedance drop in such areas are lacking.

There is an ongoing clinical trial led by Ullah et al to assess the value of LPs and AI in VT ablation (ClinicalTrials.gov Identifier: NCT03437408). This is a small (n = 15) open label study which aims to collect ventricular substrate maps (with automated tagging in different pacing modalities) in patients undergoing ablation for VT. During ablation, impedance and AI data will be collected. Thus, the only experience investigating radiofrequency (RF) lesion depth and width in normal voltage myocardium (NVM), LVM and IVM defined by electroanatomical maps (EAM) and their association with AI and impedance drop is limited to basic science studies.

Tofig et al performed in vivo EAM and endocardial RF ablation in 10 pigs at 12 weeks post‐myocardial infarction (MI) by left anterior descending artery (LAD) occlusion and reperfusion.^58^ The EAM and endocardial ablations were performed in the NVM, IVM, and LVM myocardium. Ninety RF lesions were evaluated in the NVM (n = 36), IVM (n = 32) and LVM (n = 22) groups. In terms of baseline characteristics, there was no difference in CF, power, AI and duration of RF ablation between the 3 groups. Interestingly despite this, the RF lesion depth and width were smaller in IVM and LVM compared to NVM (both *P* < .001). In addition, the RF lesion depth and width were smaller in LVM compared with IVM (*P* < .001). The RF lesion depth and width correlated with CF, AI and impedance drop in NVM and IVM: The RF lesion depth and width correlated stronger with AI in NVM than in IVM (depth *P* < .01; width *P* < .05) while the impedance drop during ablation was higher in NVM (27 ± 10Ω) compared with IVM (12 ± 7Ω, *P* < .001) and LVM (9 ± 3Ω, *P* < .001) but this did not differ between IVM and LVM. Tofig et al also utilized native contrast magnetic resonance imaging (ncMRI) to correlate the RF lesion depth and width with that of gross anatomical evaluation. They found that the RF lesion depth and width as assessed by cnMRI correlated closely with that of gross anatomical evaluation in NVM, IVM (depth and width *P* < 0.001 in both NVM and IVM) and LVM (depth and width *P* < .05).

The findings by Tofig et al suggest that IVM and LVM regions are less prone to conductive heating compared to NVM and this may be because of the higher degree of heterogeneity of myocardial composition in the IVM and LVM regions. In terms of the potential applicability in the clinical setting, the findings of this study could one day be used to guide the titration of RF delivery to ensure adequate lesion formation especially in the IVM and LVM regions.

This was not the first study evaluating RFCA in MI models but it is the first one that utilizes the newer indices to evaluate RF efficacy and differences between areas of normal myocardium and scar. A similar study was performed by Kovoor et al^59^ using a canine LAD ligation model without reperfusion investigating RF lesion size after epicardial ablation with a needle catheter; evaluating intramural temperature at different distances from the ablation site both in NVM and in the scar zone with no differences observed between them. However there are several key differences between Tofig's and Kovoor's work, such as site of ablation (endocardial vs epicardial), different catheters (3.5 mm irrigated vs needle) and the presence of scar zone with and without reperfusion after MI. Data supporting Tofig's findings also come from An et al's in vitro study using normal bovine myocardium and diseased human tissue with considerably smaller RF lesions in diseased tissue.^60^

Similar to AI, Lesion Size Index (LSI) is a multi‐parametric ablation index that incorporates in a weighted formula the 3 components contact force, power and duration of radiofrequency delivery. In an in‐vitro model of radiofrequency ablation on porcine left ventricular myocardial slabs, LSI values were highly predictive of lesion width and depth, whereas power and CF did not ^61^. More recently, in an ex‐vivo model, it has been shown that at similar LSI values, lesion width but not depth was dependent on the catheter tip orientation towards the tissue ^62^, whereas AI has been shown to be highly dependent on the angle of orientation of catheter tip, as the correlation with lesion width and size is lost at shallow contact angles ^63^. These results indicate that LSI is a reliable marker for safety and efficacy during ablation even with varying catheter to tissue contact angles.

In a study investigating tissue temperatures in porcine heart specimens during radiofrequency ablation at different power settings for fixed AI and LSI values, Takemoto et al showed that the maximum tissue temperature was significantly lower at 40 W compared to 20 W for a given LSI value, providing evidence for a safer LSI‐guided procedure at higher power RF settings. In comparison, an equivocal AI provided a similar tissue temperature at high and low power. Of note LSI‐guided lesion size was significantly larger with 20 W applications due to the longer duration of high temperature and enhanced heat distribution, whilst lesion size did not significantly alter with AI‐guided 20 W or 40 W applications for the same AI value^64^.

A few preliminary studies have assessed performance of LSI in clinical setting, showing better long‐term free survival from AF recurrence in PVI procedures guided by LSI compared contact force monitoring ^65^. More recently, Dello Russo et al attempted to identify a cut‐off value of LSI for the delivery of effective lesions during PVI. In this retrospective study of 37 patients, the authors reported that a mean LSI of 5 was associated with a lower incidence of arrhythmias recurrence at 1‐year follow up ^66^. The LIVID study (A study on Lesion Index guided VentrIcular tachycarDia ablation) is on‐going at our Institution and will assess the performance of LSI during VT ablation with the aim to provide optimal LSI targets for ablation, based on the pre‐ablation high density derived substrate map. Recently a catheter capable of measuring local myocardial impedance has been introduced into clinical practice; the IntellaNav MiFi OI Catheter (Boston Scientific, Marlborough, MA) which is equipped with three equally spaced tip microelectrodes. An alternating current injected between these microelectrodes and the distal catheter ring creates a local potential field, which is distorted at contact with myocardium and converted by an algorithm called ‘DIRECTESENSE’ into impedance. Preliminary clinical studies have shown that the absolute drop and drop rate of Local Impedance (LI) is a reliable marker of an effective lesion in both AF and VT^61^, ^67^, ^68^. Münkler et al in a mixed cohort of 28 patients undergoing VT ablation have reported that high values of absolute LI and rate of LI drop are associated with termination of VT during ablation ^68^.

The potential advantage of LI DIRECTSENSE in respect to AI and LSI is that it facilitates a substrate map based on tissue impedance defining areas of scar and normal myocardium as well areas of impedance drop after ablation. Indeed the data reported by Münkler et al in patients with NIDCM suggests a potential preference for LI guided procedure for VT ablation in this cohort of patients. Indeed it is in the context of NIDCM where VT ablation presents the most unsatisfactory outcomes due to the already described characteristics and distribution of myocardial scar. Recently, Della Bella and colleagues reported on the feasibility and efficacy of bipolar ablation in patients with NIDCM VT originating from the deep inter‐ventricular septum. Of note, the authors regulated the delivery of bipolar radiofrequency pulses on the basis of a drop in impedance of 20‐40 Ω as per prior ex vivo study protocols.

**FIGURE 2:**
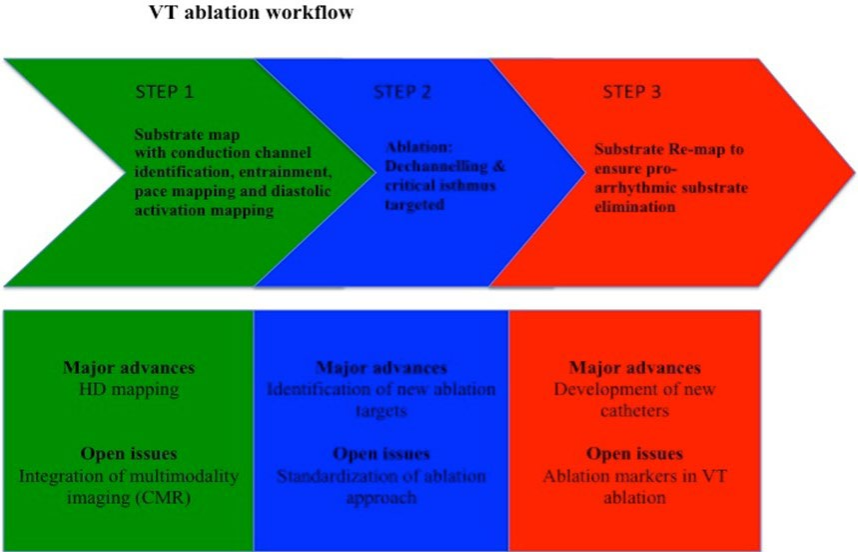
Central Illustration showing the three steps in which VT ablation can be differentiated

Increasing insights regarding radiofrequency lesions and their anatomical characteristics have come from the expanding use of cardiac MRI (CMR). Initial observations have emerged from animal models ^69^, ^70^, ^71^. Until recently, few studies had been performed aiming to identify specific CMR findings caused by ablation in human hearts with hyperenhanced (HE) lesions having been identified years after ablation of ventricular premature beat (VPB)s ^72^ and in another study early nonenhanced (NE) lesions mimicking typical no‐reflow phenomenon in acute MI after ablation of AF ^73^ possibly caused by coagulation and contraction band necrosis that hampers diffusion of gadolinium. Dinov et al. have recently published ^34^ a study in which the investigators better describe the relationship between myocardial lesion characteristics and outcomes in patients treated with ablation for VA early after the procedure. Comparing CMR examinations performed before and early (by the third day post‐ablation) post‐procedure they reported that RF lesions appear as NE dark areas surrounded by a hyperenhanced rim. They further analyzed images obtained calculating volume and depth of NE lesions showing a positive correlation between these NE lesions and impedance drop with values considerably higher in patients with transmural (>75% of LV wall) lesions. However, others have reported an alternative correlation when considering acute RFCA efficacy (no VPB in the 30 minutes post‐ablation or inability to trigger VT) showing larger lesion volume and depth as well as a larger impedance drops when procedures have failed. This may be due to the inability to reach the arrhythmia substrate. With the observation that early NE lesions evolve into scar tissue after 3 months ^73^, these lesions appear to be a more reliable marker of long term efficacy than late HE lesions as previously shown. These findings offer again the opportunity to highlight the fact that in RFCA of VA, substrate modification and LP elimination is more important for a successful procedure than lesion transmurality, emphasising the need for precision tools and techniques. (Figure 2)

In conclusion, RFCA has become a mainstream therapy for the treatment of VT. The procedure has evolved focusing on the accurate identification of targets for ablation, improving and defining substrate mapping. In addition, there has been a technological advancement in the monitoring and titration of energy delivered to yield effective RF lesion formation. However, the application of these tools have been scarcely investigated and implemented in the practice of VT ablation. Since VT recurrence in patients treated with RFCA can be related, at least partly, to inadequate RF lesion formation, it is imperative that we continue to explore the need for robust, transferrable markers of ablation efficacy.

## CONFLICTS OF INTEREST

The authors declare that there is no conflict of interest.
